# Toward an Evolved Concept of Landrace

**DOI:** 10.3389/fpls.2017.00145

**Published:** 2017-02-08

**Authors:** Francesc Casañas, Joan Simó, Joan Casals, Jaime Prohens

**Affiliations:** ^1^Fundació Miquel Agustí/BarcelonatechBarcelona, Spain; ^2^Institut de Conservació i Millora de l’Agrodiversitat Valenciana, Universitat Politècnica de ValènciaValència, Spain

**Keywords:** landraces, heirlooms, evolution, breeding, biotechnology, traditional varieties, cultural preferences

## Abstract

The term “landrace” has generally been defined as a cultivated, genetically heterogeneous variety that has evolved in a certain ecogeographical area and is therefore adapted to the edaphic and climatic conditions and to its traditional management and uses. Despite being considered by many to be inalterable, landraces have been and are in a constant state of evolution as a result of natural and artificial selection. Many landraces have disappeared from cultivation but are preserved in gene banks. Using modern selection and breeding technology tools to shape these preserved landraces together with the ones that are still cultivated is a further step in their evolution in order to preserve their agricultural significance. Adapting historical landraces to present agricultural conditions using cutting-edge breeding technology represents a challenging opportunity to use them in a modern sustainable agriculture, as an immediate return on the investment is highly unlikely. Consequently, we propose a more inclusive definition of landraces, namely that they consist of cultivated varieties that have evolved and may continue evolving, using conventional or modern breeding techniques, in traditional or new agricultural environments within a defined ecogeographical area and under the influence of the local human culture. This includes adaptation of landraces to new management systems and the unconscious or conscious selection made by farmers or breeders using available technology. In this respect, a mixed selection system might be established in which farmers and other social agents develop evolved landraces from the variability generated by public entities.

## The Concept of Landrace

Widely used in the literature, the term “landrace” encompasses a range of different concepts that have varied over time depending on prevailing trends in the use and conservation of genetic resources. After an initial period in which it was considered important to conserve landraces to maintain biodiversity, nowadays there is an increasingly promoted commercial message that holds that landraces are generally endowed with superior nutritional and sensory properties (“a flavor of the past” belief), and this has influenced the concept of landrace. For many years after von Rümker (1908) introduced the term, the term “landrace” was applied to cultivars that had evolved without conscious selection. Zeven (1998) proposed a new and more concise description of landrace, modifying Mayr’s (1938) early distinction of landrace and taking into account the possible contamination of landraces with foreign material that was neglected by Mayr (1937). According to Zeven (1998) “*an autochthonous landrace* is a landrace grown for a long period in the farming system concerned. As the environment changes annually and as the landrace becomes ‘contaminated’ with few genotypes of other landrace(s), or cultivar(s) it will continuously adapt itself” and “*an allochthonous landrace* is an autochthonous landrace of a foreign region recently introduced into the region concerned. This will be a rare type, as after its introduction it frequently becomes contaminated with a few genotypes of the autochthonous landraces or locally grown cultivar(s). Depending on the number of generations of after growth and on the frequency of seed change, it may become an autochthonous landrace.” Nevertheless, in his review Zeven (1998) considered that the amended Manholt’s definition was still the best description of landraces at that time: “an autochthonous landrace is a variety with a high capacity to tolerate biotic and abiotic stress resulting in a high yield stability and an intermediate yield level under a low input agricultural system." All these definitions are based more on the properties of the population than on the events or methods leading up to the development of the landrace or its history. It is not concerned with who has multiplied the seeds or how, or with whether conservative selection or innovative techniques are involved in the multiplication of the landrace.

More recent approaches to the concept of landrace again incorporate elements related to the mechanics of its evolution. Villa et al. (2005) define landrace as “a dynamic population of a cultivated plant that has historical origin, distinct identity and lacks formal crop improvement, as well as often being genetically diverse, locally adapted and associated with traditional farming systems.” In the Task Force on Farm Conservation and Management report, Del Greco et al. (2007) chose to use the Villa et al. (2005) definition for their future activities, thus tying the concept of landrace to traditional farming systems and the absence of formal breeding (although both these terms are ambiguous, since farmers have always used genetic and environmental selection).

Finally, in an excellent review, Negri et al. (2009) approach the subject of on-farm conservation, proposing that the selection techniques used should be eliminated from the definition of landraces: “on-farm conservation should be reformulated as the management of genetic diversity of locally developed crop varieties (landraces) by farmers within their own agricultural, horticultural or agri-silvicultural systems.” This view considers that landraces can evolve within any farming system and avoids the restriction that breeding landraces should forgo the use of genetic knowledge. The ambiguous term “traditional” is replaced with the description “their own agricultural, horticultural or agri-silvicultural systems” (i.e., with any farming system in use at a given place and time).

Zeven points out that landraces are continually evolving and also continually mixing with other landraces or cultivars on a genetic level. Natural and artificial selection together with migration resulting from the exchange of seeds can contribute to creating different landraces (populations), as well as groups of interrelated landraces (which could be considered as metapopulations). Therefore, landraces should be viewed as evolving entities in contrast to modern cultivars, which are expected to be maintained true to type according to Union for the Protection of New Varieties of Plants (UPOV) rules. In this respect, Harlan (1965, 1992) enlightenment on the role of weeds in the evolution of landraces gave rise to a dynamic view of the flow of genes among wild plants, landraces, and improved varieties. Information about gene flow has increased considerably thanks to the use of molecular markers and has also raised concerns on the potential dissemination of transgenes into landraces and wild types. The conclusion is that genes are transferred in all directions, both in allogamous and autogamous plants (Ellstrand et al., 1999, 2013; Jarvis and Hodgkin, 1999; Messeguer, 2003; Gompert and Buerkle, 2016), even though gene transfer can vary tremendously among species and populations, between plants within a population, and even over time (Ellstrand, 2014). Interestingly, after spontaneous crossing from commercial hybrids into some Italian landraces of maize, the introgressed genes have become the main targets for positive selection in farmers’ traditional management (Bitocchi et al., 2009, 2015). Causse et al. (2013) report a similar phenomenon in tomatoes. This evidence argues against attempts to bind the concept of landrace to isolated, immobile, easily classifiable cultivars that can evolve only without formal breeding (i.e., with no application of genetics knowledge) or to cultivation with techniques now considered obsolete that are rooted in ideological preconceptions. In fact, the most conservative definitions doom landraces to become artifacts in museums, since these cultivars can only evolve without formal breeding. Any definition that does not take into account technological changes relegates most landraces to the status of curios, kept in existence through on-farm conservation as a mere subsidized activity, which can be inviable under the usual restriction of funds for *in situ* conservation.

## Landraces Revisited

We propose a more inclusive definition of landraces as plant materials consisting of cultivated varieties that have evolved and may continue evolving, using conventional or modern breeding techniques, in traditional or new agricultural environments within a defined ecogeographical area and under the influence of local human culture. This includes adaptation of landraces to the management systems and the unconscious or conscious selection made by farmers or breeders with available technology. This coevolution has been modeling landraces as a result of several factors: the initial genetic variation; the generation of new variation through mutation, migration, recombination, and crossing with other populations; the soil, climate, and other ecological conditions of the region of cultivation; and the influence of humans, which includes cultivation techniques (evolving over time) and preferences (sensorial, nutritional, religious, etc.). This definition emphasizes the role of humans in the evolution of landraces because human intervention has been a key factor (in fact humans and cultivated plants have constituted a symbiotic system since the Neolithic period).

As a consequence of this coevolution, populations that have not undergone organized genetic improvement meet Zeven’s (1998) definition: high tolerance to biotic and abiotic stress prevalent in the area, medium yields, yield stability over time, and adaptation to low inputs. In fact, the key point is yield stability, which enables the system consisting of the landrace and dependant human population to extend over time. This restricted definition of landrace implies that evolution of landraces halted with the appearance of improved varieties derived with knowledge of genetics, which would mean that the history of landraces came to an end in the 20th century in most places (while everywhere else genetically improved cultivars continued to gain ground). Under this definition, landraces could only survive in isolated spots, such as amateurs’ gardens, where they would be free from genetic contamination and introgression of new foreign genes (providing that someone takes charge of multiplication to conserve the landrace). This approach threatens to transform landraces into something they have never been: museum relics.

It is an undeniable fact that genetically improved varieties have become predominant throughout most of the world. This predominance has been made possible by high energy inputs in crop production (machinery, protection from pests, fertilizers, etc.) and heavy investment in research and genetic improvement. To the extent that these factors molded similar environments, the same or only slightly different varieties could be cultivated in more places. With the loss of farmer specialization in propagating their landraces, professional breeders can obtain returns on investments from developing new varieties. Legislation on protection of cultivars and seed trade also favor improved cultivars by demanding homogeneous materials for each variety commercialized. However, the main reason for genetic erosion is the inability of unimproved landraces to compete against improved varieties. Cultivated germplasm has evolved since Neolithic times, so it should not surprise us that this has continued into the modern times; and in some cases it has sped up dramatically as in the development of improved varieties for widespread use.

Nevertheless, these improved varieties have drawbacks: (a) cultural ties with consumers are weak or non-existent (especially important in fruit and vegetables), (b) growing them requires large investments in energy (the extreme case being high-tech greenhouses), and (c) methods of distribution (e.g., harvesting unripe fruit) can lower sensorial quality. On the other hand, materials that meet the narrow definition of landraces also suffer from serious drawbacks such as low yields and vulnerability to new pests introduced in new areas as a consequence of globalization.

Given our society’s growing concern for the environment (inputs, recycling, pesticides, etc.) and growing interest in identity issues (sensorial value of food, local and ethnic cuisine, etc.), the ecological aspects of traditional varieties (aspects related to the milieu) can represent values appreciated by consumers and therefore be used as tools for development and progress by farmers. Thus, there is a need for a less orthodox conceptualization of landraces, one that is based on values that continue to be interesting in modern societies, such as adaptation to the environment, need of reduced inputs, cultural values, and diversity as a consequence of local adaptation, etc.

## Landraces, The New Generation

Our proposal aims to modify the concept of landrace delineated by Zeven (1998), while taking into account that the tools now available for selection are far more powerful than those available in the Neolithic or in the late 19th century. If we wish to continue enjoying cultivars with cultural pedigrees that are intimately associated with specific geographical areas (particular environmental traits) and thus require fewer inputs for their cultivation, the generalist strategy used in modern breeding programs is insufficient (although it will surely continue to supply a large proportion of our alimentary needs). This does not mean that we need to invent a new approach. Rather, we need only develop cultivars that meet Zeven’s criteria for landraces, using the tools now available for genetic improvement, agronomics, and biotechnology, which is nothing more than a new generation of landraces for a new period of Agriculture.

Given that adaptability to particular environments, general resilience, cultural value, tolerance to local stresses, sensorial value, etc. are traits often controlled by multiple genes, the most reasonable material for initial development of new generations of local cultivars consists of historical landraces, found in seed banks or under local cultivation.

Our proposal involves extending the concept of landraces beyond their currently recognized value as a reservoir of genes. We propose to use these materials, study their potential, and correct their defects with available technology. The first wave of transformations that candidate landraces might undergo are the introduction of monogenic traits, such as resistance to pathogens, although this might create a “boom and bust” phenomenon. Globalization has allowed pests and pathogens to circulate freely, so farmers cultivating historical landraces often have to apply many phytosanitary treatments or risk losing their entire crop, making it impossible to meet the goals of keeping inputs to a minimum and using minimally aggressive agricultural practices. Problems related to traditional methods, such as linkage drag in backcrossing (introducing undesirable genes linked to the gene introduced mainly when the donor is a wild plant), can be overcome by biotechnological approaches available today (Nogué et al., 2016) and others that will surely be devised in the future.

On a small scale, and still fundamentally through backcrossing or classical selection, new cultivars have already been obtained from landraces. In Spain, for example, with this aim of local and cultural adaptation, new improved materials have been developed from landraces in tomatoes (Casals et al., 2010; Garcia-Martínez et al., 2011), beans (Bosch et al., 1998; Almirall et al., 2010; Ferreira et al., 2012), onions (Simó et al., 2012), and eggplants (Prohens et al., 2009). It would be difficult to argue that these cultivars should not be considered landraces because they have been obtained using breeding techniques like directed crossings and/or self-fertilization followed by selection.

In this new view, intravarietal heterogeneity can be replaced by a large number of landraces with different traits that fit in together with the different environmental characteristics encompassed in the local cultural sphere. For example, different landraces with similar sensory profiles and yields, adapted to the needs of local cuisine, but with different degrees of earliness to allow scaled sowing or to allow for yearly variations in the weather, especially in moisture content of the soil for sowing, might be developed. Thus, we could even consider using landrace hybrids, whether between two landraces or between a landrace and a single inbred line.

## Evolution in Landraces Nowadays

**FIGURE 1:**
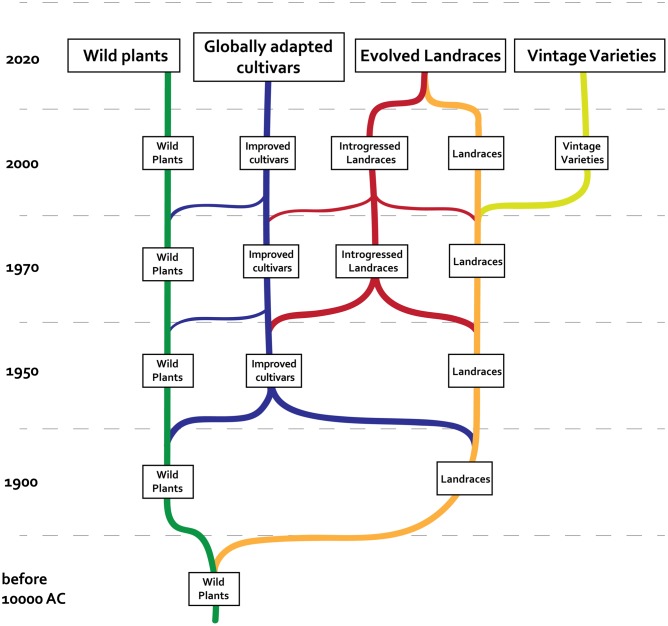
**Timeline of historical relationship among different kinds of plant materials and prospects for the immediate future**.

Before the advent of scientific breeding techniques, landraces evolved together with the farmers that managed them within their cultures (including aesthetic preferences, ethical values, and technological knowledge) in the soil and climatic conditions in which the evolutionary process occurred. Large-scale breeding and better control over environmental factors, together with the availability of vast amounts of energy and global food distribution systems, have transformed landraces into new types which generally can be classified in different categories: (a) generalized, universally embraced, commercial cultivars of extremely homogeneous materials that are no longer bound to a particular local culture, which are managed by breeding corporations, (b) cultivars that we continue to refer to as landraces, which include a blend of “unselected” varieties that have continued to evolve “on farm,” but that are usually heavily introgressed from genetically improved varieties or other “landraces” with which they have been crossed, (c) “unselected” landraces, which in some cases have remained more or less free of introgression, in which evolution has been suspended by conservative selection (heirlooms or vintage varieties in the strictest sense), and (d) varieties that maintain their character as garden varieties, easily confused with the “landraces” described in (b). Thus, the evolution of landraces can take different paths, some of which lead to products that conserve the spirit of landraces before the advent of scientific selection (**Figure 1**).

If we wish to continue the coevolution of landraces on a local level with strong ties to local culture (what we understand to be their differential trait), we need to manage this coevolution. This task cannot be relegated to farmers dedicated to cultivating landraces with traditional techniques in relatively isolated sites. This unrealistic vision is rooted in a conservative ideology that does not take evolution into account. We need to design participative strategies in which, just as society has moved toward a division of labor with increasing specialization, various social agents participate in the evolution of the landraces. The burden of this task cannot be placed only on farmers, because farmers have also become specialists with their own niche in our knowledge-based societies that tend to distribute responsibilities among various agents. In this respect, participatory plant breeding (PPB), preferably implemented through public-private partnerships (PPP), could play an important role in the evolution of landraces in order to respond to the challenges and demands of the different stakeholders in the marketing chain going from producer to consumer.

**FIGURE 2:**
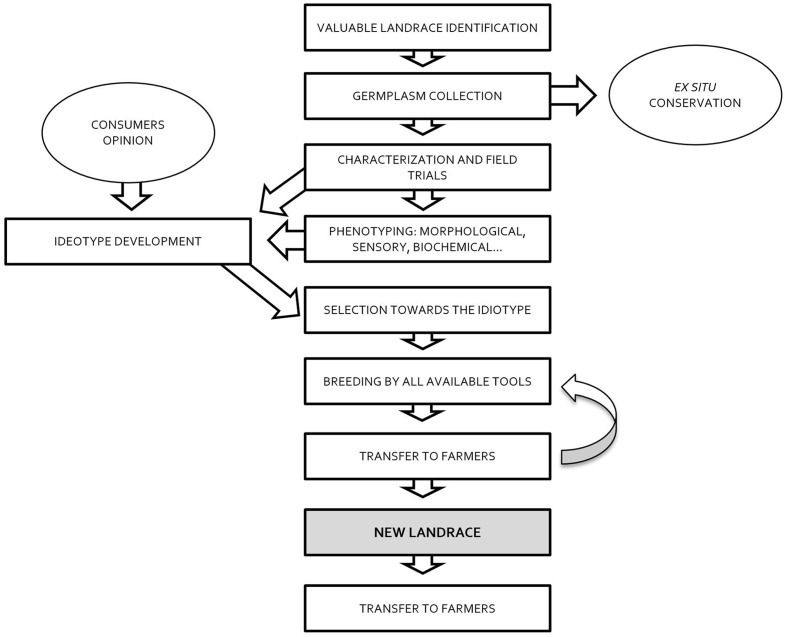
**The approach to creating evolved landraces using the present available tools**.

The first step in managing the evolution of landraces should be filtering by the local cultures and societies that are the depositories of these materials. These societies should evaluate the plant genetic resources linked to their histories that are extant *in situ* or *ex situ*. It is important to identify varieties with traits that have added value in today’s society (nutritional, nutraceutical, sensorial, and/or cultural value), recognizing that the adaptation to the local environment is already the defining characteristic of these materials (**Figure 2**). Territories without local varieties should be able to seek out landraces from other territories that would become allochthonous according to Zeven’s (1998) definition. New landraces, developed according to the needs of societies, could be created. In fact, allochthonous landraces have been developed throughout history, and if we consider the strictest definition of landrace to include only autochthonous materials, these could only exist in territories where wild types were domesticated.

The second step is to evaluate the landraces’ potential, considering rational cultivation techniques used in a certain place and time (**Figure 2**). This merely means using cultivation techniques that seem most reasonable and accepted by a given society. Nowadays in advanced Western societies, this means low-input agriculture, with minimal agrochemical intervention. In the future, other approaches might be considered more rational. Based on this assessment, technology centers and experimental stations should be put in charge of characterizing the materials and determining their commercial potential in close collaboration with farmers, who need to be involved in these processes.

The third step is to correct the varieties’ weaknesses without undermining their strengths (**Figure 2**). In our opinion it is essential to take full advantage of all available tools from classic breeding and biotechnology. As stated above, landraces should be defined by their essence, not by the methods used to obtain them. This approach requires the intervention of institutions that generate unselected materials, introducing variability for the traits that need to be modified. Again, farmers must serve as the final filter for a large number of new candidate varieties that technological centers and experimental stations can provide.

One last point that must be resolved is the ownership of the landraces, their certification, and their maintenance until they are replaced by newly evolved types (it is to be expected that landraces will have a certain half-life during which they will be maintained invariable through conservative selection, while new, further-evolved versions are being developed) (**Figure 2**). Dealing with this point requires imagination and generosity. Registries of conservation varieties represent an attempt to provide a legal framework for these materials, but further developments are needed to take into account the inherent heterogeneity of landraces.

The review of Negri et al. (2009) warns that “Unless action is taken immediately, landraces losses will continue and complete extinction is the only possible conclusion. As a consequence, urgent action is required to inventory, rescue, and preserve the wealth of European landrace diversity. The first logical step appears to be to compile inventories, later threat assessment and prioritization for conservation should be carried out.” We believe that this paradigm of fear that is usually linked to the idea of landraces as a reservoir for genes to improve commercial varieties so that they can face the challenges of the future should be supplanted by a more historical vision that considers the evolution of the landraces, preserving the spirit of the concept while increasing diversity with truly competitive materials. In other words, we propose preserving landrace diversity through their use in multiple, objectively compatible plant materials.

## Author Contributions

FC and JP conceived the project and supervised the manuscript. FC, JS, JC, and JP drafted the manuscript and contributed to discussions. JS and JC designed and drew the figures.

## Conflict of Interest Statement

The authors declare that the research was conducted in the absence of any commercial or financial relationships that could be construed as a potential conflict of interest.

## References

[B1] Almirall A. R. Bosch L. Romero Del Castillo M. Rivera A. Casañas F. (2010). “Croscat” common bean (*Phaseolus vulgaris* L.), a prototypical cultivar within the “Tavella Brisa” type. *HortScience* 45 432–433.

[B2] Bitocchi E. Bellucci E. Rau D. Albertini E. Rodriguez M. Veronesi F. (2015). European flint landraces grown in situ reveal adaptive introgression from modern maize. *PLoS ONE* 10:e0121381 10.1371/journal.pone.0121381 PMC4390310 25853809

[B3] Bitocchi E. Nanni L. Rossi M. Rau D. Bellucci E. Giardini A. (2009). Introgression from modern hybrid varieties into landrace populations of maize (*Zea mays* ssp. mays L.) in central Italy. *Mol. Ecol.* 18 603–621. 10.1111/j.1365-294X.2008.04064.x 19215582

[B4] Bosch L. Casañas F. Sánchez E. Pujolà M. Nuez F. (1998). Selection L67, a pure line with true seed type of the Ganxet common bean (*Phaseolus vulgaris* L.). *HortScience* 33 905–906.

[B5] Casals J. Bosch L. Casañas F. Cebolla J. Nuez F. (2010). Montgrí, a cultivar within the montserrat tomato type. *Hortscience* 45 1885–1886.

[B6] Causse M. Desplat N. Pascual L. Le Paslier M. C. Sauvage C. Bauchet G. (2013). Whole genome resequencing in tomato reveals variation associated with introgression and breeding events. *BMC Genomics* 14:791 10.1186/1471-2164-14-791 PMC4046683 24228636

[B7] Del Greco A. Negri V. Maxted N. compilers (2007). “Report of a task force on on-farm conservation and management,” in *Proceedings of the Second Meeting, Stegelitz, Germany*, (Rome: Bioversity International), 19–20.

[B8] Ellstrand N. C. (2014). Is gene flow the most important evolutionary force in plants? *Am. J. Bot.* 101 737–753. 10.3732/ajb.1400024 24752890

[B9] Ellstrand N. C. Meirmans P. Rong J. Bartsch D. Ghosh A. de Jong T. J. (2013). Introgression of crop alleles into wild or weedy populations. *Annu. Rev. Ecol. Evol. Syst.* 44 325–345. 10.1146/annurev-ecolsys-110512-135840

[B10] Ellstrand N. C. Prentice H. C. Hancock J. F. (1999). Gene flow and introgression from domesticated plants into their wild relatives. *Annu. Rev. Ecol. Evol. Syst.* 30 539–563. 10.1146/annurev.ecolsys.30.1.539

[B11] Ferreira J. J. Campa A. Pérez-Vega E. Rodríguez-Suárez C. Giraldez R. (2012). Introgression and pyramiding into common bean market class fabada of genes conferring resistance to anthracnose and potyvirus. *Theor. Appl. Genet.* 124 777–788. 10.1007/s00122-011-1746-x 22146986

[B12] Garcia-Martínez S. Grau A. Aranzazu A. Rubio F. Valero M. Ruiz J. J. (2011). UMH 1200, a breeding line within the muchamiel tomato type resistant to three viruses. *HortScience* 46 1054–1055.

[B13] Gompert Z. Buerkle C. A. (2016). What, if anything, are hybrids: enduring truths and challenges associated with population structure and gene flow. *Evol. Appl.* 9 909–923. 10.1111/eva.12380 27468308 PMC4947152

[B14] Harlan J. R. (1965). The possible role of weed races in the evolution of cultivated plants. *Euphytica* 14 173–176. 10.1007/BF00038984

[B15] Harlan J. R. (1992). *Crops & Man*, Second Edn. Madison, WI: Crop Science Society of America and American Society of Agronomy.

[B16] Jarvis D. I. Hodgkin T. (1999). Wild relatives and crop cultivars: detecting natural introgression and farmer selection of new genetic combinations in agroecosystems. *Mol. Ecol.* 8 159–173. 10.1046/j.1365-294X.1999.00799.x 9919705

[B17] Mayr E. (1937). Alpine Landsorten in ihrer Bedeutung für die praktische Züchtung. *Forschungsdienst* 4 162–166.

[B18] Messeguer J. (2003). Gene flow assessment in transgenic plants. *Plant Cell Tissue Organ. Cult.* 73 201–212. 10.1023/A:1023007606621

[B19] Negri V. Maxted N. Veteläinen M. (2009). “European landrace conservation: an introduction,” in *European Landraces: on farm Conservation, Management and Use: Biodiversity Technical Bulletin no 15*, eds Veteläinen, M. Negri V. Maxted N. (Rome: European Cooperative Programme for Plant Genetic Resources).

[B20] Nogué F. Mara K. Collonnier C. Casacuberta J. M. (2016). Genome engineering and plant breeding: impact on trait discovery and development. *Plant Cell. Rep.* 35 1475–1486. 10.1007/s00299-016-1993-z 27193593 PMC4903109

[B21] Prohens J. Muñoz-Falcon J. E. Rodriguez-Berruezo A. Ribas F. Castro A. Nuez F. (2009). ‘H15’, an Almagro-type eggplant with high yield and reduced prickliness. *Hortscience* 44 2017–2019.

[B22] Simó J. Romero Del Castillo R. Almirall A. Casañas F. (2012). “Roquerola” and “Montferri” first improved onion (Allium cepa L.) cultivars for “calçots” production. *HortScience* 47 801–802.

[B23] Villa T. C. Maxted N. Scholten M. A. Ford-Lloyd B. V. (2005). Defining and identifying crop landraces. *Plant Genet. Res.* 3 373–384. 10.1079/PGR200591

[B24] von Rümker K. (1908). Die systematische Einteilung und Benennung der Getreidesorten für praktische Zwecke. *Jahrb. Dtsch. Landwirtsch. Ges.* 23 137–167.

[B25] Zeven A. C. (1998). Landraces: a review of definitions and classifications. *Euphytica* 104 127–139. 10.1023/A:1018683119237

